# Pulsed Dye Laser Does Not Seem as Effective as Red Light in Basal Cell Carcinoma Mal-Pdt: A Small Pilot Study

**DOI:** 10.1155/2012/396481

**Published:** 2012-10-31

**Authors:** M. Fernández-Guarino, A. Harto, P. Jaén

**Affiliations:** Departament of Dermatology, Alcalá de Henares University, Carretera de Colmenar Km 9,100, 28034 Madrid, Spain

## Abstract

Multiple light sources can be used for photodynamic therapy (PDT) with good results, but there are few comparative studies. This study compares the efficacy of treatment of basal cell carcinoma with PDT and two light sources, the non-coherent red light and pulsed dye laser 595 nm. In this small pilot study red light is more effective, but many more studies are needed to draw definitive conclusions.

A variety of light sources, coherent and not coherent, have been demonstrated efficacy in photodynamic therapy (PDT) but there is an absence of comparative studies in clinical practice. Red light LED is probably the most used for conventional PDT [1] and pulsed dye laser 595 nm (PDL) is among the coherent light the most appropriate for PDT although its parameters have not been well defined [2]. Our objective was to compare both light sources in methylaminolevulinic acid (MAL) PDT for superficial basal cell carcinoma (BCC).

A small pilot study was performed selecting superficial histologically confirmed BCC with a diameter greater than 3 centimeters. One half of the lesion was treated with conventional PDT, MAL occluded 3 hours, and red light (630 nm 37 J/cm^2^, 8 minutes, Aktilite) and the other half of the lesion was treated with MAL occluded 3 hours and PDL 595 nm ( 7 mm, 6 ms, overlap 50%, three passes, Candela Vbeam). A total of 2 sessions one week apart were applied and clinical response was assessed at 0, 3, and 6 months after the treatment. Previous to treatment clinical and fluorescence photographs were taken and tolerance in each half was evaluated in an analogue scale by the patient (from 0 to 10). At first, the order of the application of each light would be randomized, but when red light was applied only in one half of the BCC the whole lesion photobleached, so PDL was applied always first in order to evaluate fluorescence diagnosis.

A total of 6 patients and 6 BCCs were treated (see Table 1). The average diameter of the lesions was 3.5 cm (range 3-4). Five of the patients achieved complete response with red light and incomplete with PDL.  Figure 1 shows an image of a basal cell carcinoma treated with red light in a half and laser in the other half. Patient number 3 did not response to any treatment. Fluorescence pretreatment was always positive, but only photobleached in the half treated with red light. The half treated with PDL, after red light in the other half, also photobleached, but not after PDL illumination. Tolerance was better with red light, with a mean of 4 points (range 2–9) than with PDL with a mean of 7,5 points (range 3–10).

In the literature reviewed lasers have been suggested to be as effective as no coherent light sources in PDT of BCC [1, 2], but there are few publications with this modality of PDT and an absence of comparative well-designed studies [3, 4]. Calzavara-Pinton published in 1995 good results in ALA-PDT with a 630 nm dye laser (60 J/cm^2^) [3] and Clark in 2003 obtained similar results to broadband light sources with a 630 nm diode laser (125 J/cm^2^) in a descriptive study also with ALA-PDT [4]. Neodymium: Yag laser 630 nm has also been used for ALA-PDT of BCC with good results [1]. This is the first study with MAL-PDT in BCC and the first with a half-half design.

The main limitation of these studies is the impossibility to compare different light sources and also different photosensitizers. Laser and noncoherent light sources, including LEDs, have been shown efficacy in BCC-PDT but they have different dosimetric considerations because they have fundamental differences in their characteristics. In this way, a recent study proposed a protoporphyrin IX (PpIX) index which allows measuring the spectral characteristics of the light sources used and comparing them [4].

Non coherent lights are easy to operate, stable, not expensive, require little maintenance, and provide a wide area of illumination. Laser lights require less time of exposure but they are expensive, not always available, and need expertise to use. Theoretically, pulsed lasers allow oxygen repletion in the treated tissue between each pulse but in a recent study, Strasswinner concluded that PDL is capable of activating PDT but produces dramatically less PDT reaction than the standard blue broadband light [5].

We used PDL with those parameters in order to avoid purpura and vascular effects but LED red light was more effective and better tolerated. All of the lesions treated were superficial histologically confirmed BCC, but other possible predictors of response to PDT as tumor thickness and size of lesion could not be assessed because of the small number of BCC included in this study. We did not continue treating more patients because of the bad results. However, further controlled studies with a higher number of patients are necessary to clarify the effect of the light sources in BCC-PDT.

## Figures and Tables

**Figure 1:**
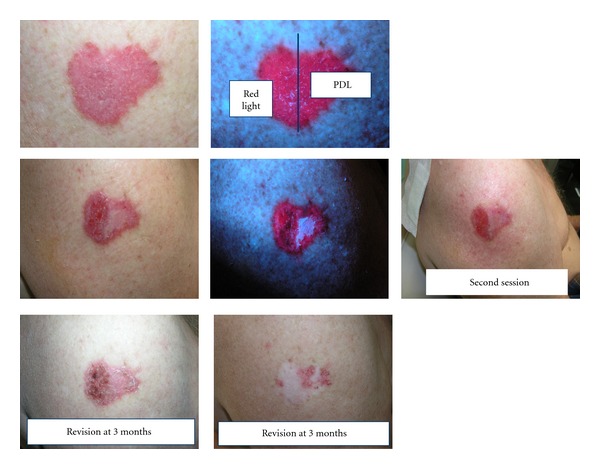
A basal cell carcinoma treated with PDT. The right half was treated with PDL and the left half with red light. Previous to the second session less inflammation was observed in the right half. A week after treatment less crustal formation appeared in the left half. After three months partial response is showed in the half treated with PDL.

**Table 1:** Summary of the patients and treatment outcome.

BCC/patient		1	2	3	4	5	6
Red light	Fluorescence pre-treatment	+	+	+	+	+	+
	Fluorescence post-treatment	−	−	−	−	−	−
	Tolerance	5	9	2	2	3	3
	Response	CR	CR	IR	CR	CR	CR

PDL	Fluorescence pre-treatment	+	+	+	+	+	+
	Fluorescence post-treatment	+	+	+	+	+	+
	Tolerance	8	10	3	8	8	8
	Response	IR	IR	IR	IR	IR	IR

CR: complete response; IR: incomplete response.

## References

[1] Morton CA, McKenna KE, Rhodes LE (2008). Guidelines for topical photodynamic therapy: update. *British Journal of Dermatology*.

[2] Choudhary S, Tang J, Elsaie ML, Nouri K (2011). Lasers in the treatment of nonmelanoma skin cancer. *Dermatologic Surgery*.

[3] Calzavara-Pinton PG (1995). Repetitive photodynamic therapy with topical *δ*-aminolaevulinic acid as an appropriate approach to the routine treatment of superficial non-melanoma skin tumours. *Journal of Photochemistry and Photobiology B*.

[4] Clark C, Bryden A, Dawe R, Moseley H, Ferguson J, Ibbotson SH (2003). Topical 5-aminolaevulinic acid photodynamic therapy for cutaneous lesions: outcome and comparison of light sources. *Photodermatology Photoimmunology and Photomedicine*.

[5] Sayre RM, Dowdy JC, Gottschalk RW (2011). Comparative effectiveness of clinically used light sources for cutaneous protoporphyrin IX-based photodynamic therapy. *Journal of Cosmetic and Laser Therapy*.

